# Coronary Intravascular Lithotripsy Effectiveness and Safety in a Real-World Cohort

**DOI:** 10.3390/jpm14040438

**Published:** 2024-04-22

**Authors:** Catarina Oliveira, Marta Vilela, Miguel Nobre Menezes, João Silva Marques, Cláudia Moreira Jorge, Tiago Rodrigues, José Almeida Duarte, José Marques da Costa, Pedro Carrilho Ferreira, Ana Rita Francisco, Pedro Pinto Cardoso, Fausto J. Pinto

**Affiliations:** 1 Serviço de Cardiologia, Departamento de Coração e Vasos, CHULN Hospital de Santa Maria, Av Prof. Egas Moniz, 1649-028 Lisboa, Portugal; 2 Structural and Coronary Heart Disease Unit, Centro Cardiovascular da Universidade de Lisboa (CCUL@RISE), Faculdade de Medicina, Universidade de Lisboa, Av Prof. Egas Moniz, 1649-028 Lisboa, Portugal

**Keywords:** calcified coronary artery disease, intravascular coronary lithotripsy, left main artery disease, stent restenosis

## Abstract

**Background**: Coronary artery calcification is a predictor of adverse outcomes after percutaneous coronary intervention (PCI). Intravascular lithotripsy (IVL) is a promising tool for the treatment of calcified lesions. The aim of this study was to assess the effectiveness and safety of IVL. **Methods**: A single-center observational study of PCI procedure, with assessment of the outcomes of patients undergoing PCI using IVL, was performed. Angiographic procedural success was used as the primary effectiveness endpoint. The primary safety endpoint was defined as a composite of cardiac death, myocardial infarction and target vessel revascularization within 30 days. **Results**: A total of 111 patients were included. Indications for PCI spanned the spectrum of chronic (53.2%) and acute coronary syndromes (43%). Lesion preparation before IVL was performed with non-compliant (42%), cutting or OPN (14.4%) balloons and with atherectomy techniques in 11% of procedures. Intravascular imaging was used in 21.6% of procedures. The primary effectiveness endpoint was achieved in 100% and the primary safety endpoint in 3.6% of procedures. Peri-procedural complications were minimal and successfully resolved. **Conclusions**: IVL was an effective and safe technique for the treatment of calcified coronary lesions. These findings contribute to the growing body of evidence supporting the use of IVL in the management of these challenging scenarios.

## 1. Introduction

Coronary artery calcification is reported in 18% to 31% of percutaneous procedures [1,2] and its prevalence is set to increase with the growing relevance of aging, diabetes, arterial hypertension and chronic kidney disease [3,4]. Calcification is known to reduce arterial compliance and compromise both short- and long-term outcomes following revascularization, increasing the risk of stent thrombosis and restenosis [1,4], mainly due to unsuccessful percutaneous coronary intervention (PCI) and stent underexpansion and malapposition [1,4,5]. 

Techniques for modifying coronary artery calcification include non-compliant, cutting and scoring balloons as well as atherectomy technologies, but they all present limitations [1,4]. Non-compliant, scoring and cutting balloons, even at high pressure inflation, may be unable to induce calcium fracture and in the presence of eccentric lesions, may be biased toward noncalcified segments [1,5,6]. Rotational and orbital atherectomy, although highly effective in lesion crossing, may result in guidewire bias [1,5,6]. All of these techniques can also lead to several peri-procedural complications, such as dissection, perforation, slow flow, no flow and peri-procedural myocardial infarction (MI) [1,4]. 

Intravascular lithotripsy (IVL), an acoustic pressure waves-based technique, has emerged as a useful tool to treat calcified lesions [1]. IVL creates controlled multiplane micro/macro fractures in the calcified plaque, improving stent expansion. Additionally, because it only requires low pressure balloon inflation, it may also further reduce the risk of complications [3]. It is thus yet another tool for better personalizing revascularization techniques according to patient characteristics. 

PCI of calcified coronary artery disease (CAD) is increasingly common and associated with higher procedural risk and risk of adverse events [7,8]. With multiple therapeutic modalities and the use of optimal technique, greater procedural success can be achieved with lower risk of complications [7]. Although all these plaque modification tools can be used separately, they are usually used together and are complementary tools to achieve optimal results in calcified lesions [7]. The decision of which technique or set of techniques to use is mainly guided by information provided by intracoronary imaging with optical coherence tomography (OCT) or intravascular ultrasound (IVUS), as calcium extension and thickness, as well as by the ability to cross the lesion [8,9,10]. With these modalities it is possible to assess plaque composition (calcification, lipid-rich plaque) and distribution and identify the need for more aggressive (rotational atherectomy, cutting or scoring balloons to induce calcium fractures) or less aggressive (direct stenting to avoid lipid embolization) lesion preparation, and facilitate choice of stent size (diameter and length) [9,10,11].

The safety and effectiveness of IVL have been reported across several clinical studies involving severely calcified coronary artery disease [1], but the evidence supporting this approach remains far less extensive than that of older techniques. Hence, in further studies, namely external validation of real-world data is necessary. In this study, we aimed to study the safety and effectiveness of IVL in a real-world cohort. 

## 2. Methods

Study design and objectives: A prospective single-center, single-arm, observational study of consecutive patients submitted to percutaneous coronary intervention (PCI) using the coronary IVL system was performed. We aimed to assess the safety and effectiveness of IVL.

All patients submitted to PCI with IVL technique were included, regardless of clinical presentation. Patients were required to have one or more target lesion with a percentage diameter stenosis ≥ 70% (or ≥50% in the left main) by visual estimation. IVL was used according to the operator’s discretion. Calcium lesion characteristics leading to IVL use were solely limited to the operator’s discretion and not to predefined intracoronary imaging criteria. Other calcium modification tools were used in addition to IVL and no head-to-head comparison was conducted. Patients provided written informed consent to the procedure and to the use of their medical information for research purposes. 

Study device: The coronary IVL catheter (Shockwave Medical, Inc., Santa Clara, CA, USA) is a single-use, sterile catheter that contains lithotripsy emitters inside an angioplasty-like balloon and is used over a regular 0.014 mm coronary guide-wire. The balloon is placed inside the target vessel and positioned over the target lesion, using the marker bands as guides. This catheter is then connected to the generator that is programmed to deliver 10 pulses at a frequency of 1 pulse/second. The IVL catheters used had a maximum of 80 pulses. 

Study endpoints: Procedural success, defined as angiographic residual stenosis <30% and TIMI III flow at the end of the procedure, was used as the primary effectiveness endpoint. The primary safety endpoint was 30-day major adverse cardiovascular events (MACE), defined as a composite incidence of cardiac death, myocardial infarction (MI) and target vessel revascularization within 30 days. Secondary endpoints included procedural success, peri-procedural complications (dissection, perforation, abrupt vessel closure and slow flow or no reflow), 30-day cardiac death, 30-day MI and 30-day target vessel revascularization. 

MI was defined according to the fourth universal definition of myocardial infarction [12]. 

Statistical analysis: Continuous variables are described as mean ± SD and categorical variables as proportions. Statistical analyses were performed using SPSS 28.0 (SPSS, Inc., Chicago, IL, USA).

## 3. Results

From March 2021 to November 2023, 111 patients were submitted to PCI using IVL as a calcium-modifying technique. Baseline clinical and angiographic characteristics are described in Table 1 and Table 2. Most patients were male, with a medium age of 73 ± 10 years old and a high prevalence of cardiovascular risk factors. Previous CAD was a frequent diagnosis, with 49% of patients with previous PCI and 17 patients with coronary artery bypass grafts.

Although chronic coronary disease was the most frequent indication for coronary angiography (53%), 43% of patients presented with an acute coronary syndrome (Table 1). 

**Figure 1:**
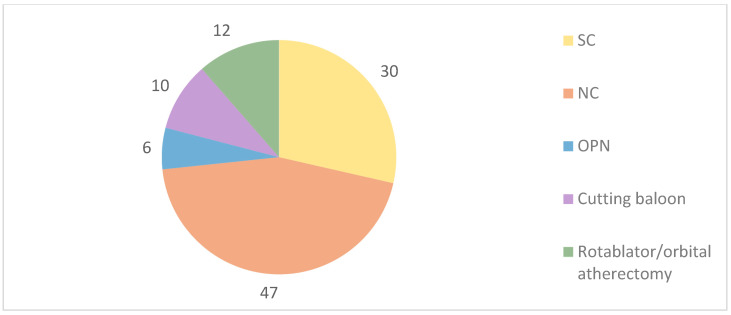
Calcified lesion preparation before IVL use. SC: semi-compliant balloons; NC: non-compliant balloons.

Regarding the technical aspects of the procedure (Table 2 and Table 3), CAD complexity was reflected by a high percentage of femoral access (37%; our centers’ average radial access is 90%) as well as by 40% of multivessel disease, 18% of stent restenosis, 12% of chronic total occlusions (CTO) and 8.1% of bifurcation lesions, five of them with significant side branch involvement. Target lesion pre-dilatation was performed with techniques other than IVL in 95.5% of the procedures, with more than one calcium modifying technique used in some patients (Figure 1). Almost all patients had only one vessel treated with IVL and one IVL balloon used, with an average of 80 pulses per patient. Balloon post-dilatation with standard non-compliant balloons was performed after IVL and before stent deployment in 38.7% of cases and following stent implantation in 57.7%. Stent delivery was performed in 93.7% of PCI and drug-eluting balloon (DEB) was used in 6.3%. Although the IVL balloon was applied mainly before stent delivery, in 6.3% of cases IVL was used to achieve optimal stent expansion after its implantation. 

PCI was guided by intracoronary imaging in 22 patients, 86% of them with intravascular ultrasound (IVUS) and 14% with optical coherence tomography (OCT). 

Importantly, 3.6% of the procedures corresponded to previous unsuccessful PCI using other calcium-modifying techniques. 

Primary safety and effectiveness endpoints: The primary effectiveness endpoint was achieved in 100% of cases. The primary safety endpoint incidence was 3.6%, driven by non-Q wave MI and target vessel revascularization (0.9%) due to stent thrombosis and by cardiovascular death (2.7%) (Table 4).

Secondary endpoints: Secondary endpoints are described in Table 4. Procedural success was achieved in 100% of cases. Peri-procedural complications after IVL balloon use occurred in 3.6% of procedures, with minor dissection in all cases—no major complications were reported. All cases were resolved following stent implantation. In all cases, the dissection occurred after the use of both IVL and non-compliant balloons and in one of them rotational atherectomy was also used before IVL. 

In-hospital cardiac death occurred in three patients, two of them due to cardiogenic shock (clinical presentation prior to PCI) and the other following an acute ischemic cerebrovascular event. Only one MI (0.9%) was reported at 30 days, due to stent thrombosis, resolved with balloon angioplasty.

Specific clinical subsets: Left main (LM) PCI using IVL was performed in nine patients, six of them in a chronic setting. Excluding one patient with ST-segment elevation MI (STEMI) with cardio-respiratory arrest at presentation, the remaining eight cases were hemodynamically stable throughout PCI. Comparing to IVL use in other vessels, fewer impulses were delivered (median of 46 pulses). An immediate procedural success was achieved in all cases, with one cardiovascular death within 24 h of the procedure due to refractory cardiogenic shock.

A hybrid procedure using IVL and atherectomy techniques was performed in 12 cases. Seven patients presented with chronic coronary syndrome (one of them with a CTO), four with non-ST-segment elevation MI (NSTEMI) and one with STEMI. Rotational atherectomy was used before IVL in 92% of the procedures, after an unsuccessful attempt to cross small-diameter balloons. In one of the cases, after IVL use, there was high-pressure balloon underexpansion and rotational atherectomy was used. One olive was used with nine patients and two in two patients (1.25 mm: six patients; 1.5 mm: six patients; 1.75 mm: two patients; 2 mm: one patient). No major peri-procedural complications were observed. 

## 4. Discussion

The data from this observational study reinforces the safety and effectiveness of PCI using IVL. The primary effectiveness endpoint was 100%. The 30-day MACE rate was very low and mainly driven by cardiovascular death not related to the procedure itself. Peri-procedural complications were both rare and minor—all were resolved after stent deployment.

The safety and effectiveness of IVL were assessed in the Disrupt CAD clinical trials (I–IV), a series of individual single-arm, prospective, non-randomized studies, that demonstrated high rates of device and procedure success, providing safety evidence in treating calcified lesions in 628 patients presenting with stable or unstable angina or silent ischemia [3,4,6,13,14,15]. All Disrupt CAD studies used similar endpoints definitions (primary safety endpoint defined as major adverse cardiac events such as cardiac death, myocardial infarction or target vessel revascularization, and primary effectiveness endpoint defined as a stenosis < 50% after stenting) and 30-day follow-up events analysis [4,6]. The primary safety endpoint occurred in 5%, 5.8%, 7.8% and 6.3% of patients in Disrupt CAD I, II, III and IV, respectively, while the primary effectiveness endpoint was achieved in more than 92% of patients in all studies [6,13,14,15,16]. In the pooled analysis of all Disrupt CAD trials [3,4], the primary safety (30-day composite occurrence of MACE—cardiac death, MI or target vessel revascularization) and effectiveness (procedural success, defined as stent delivery with residual in-stent stenosis < 30% as assessed by the angiographic core laboratory and without in-hospital MACE) endpoints were achieved in 7.3% and 92.4% of patients, respectively. At 30 days, the rates of target lesion failure (TLF), cardiac death and stent thrombosis were 7.2%, 0.5% and 0.8%, respectively [4]. These findings were consistent across all four Disrupt CAD studies [4]. Our results are in agreement with [4]. In our study, the primary safety endpoint occurred in 3.6% of cases and the primary effectiveness endpoint was achieved in all cases. Importantly, a high proportion of patients with myocardial infarction was included, contrary to the Disrupt CAD studies [4,6,13,15,16], which excluded these patients. Therefore, our study adds new and important data supporting this technique in the full spectrum of coronary syndromes presentation.

Severely calcified lesions are the biggest challenge in PCI, as they may limit the crossing of lesions, preclude adequate pre-dilation with balloon angioplasty, interfere with optimal stent expansion and also lead to an increased risk of peri-procedural complications like dissection and abrupt vessel closure [3,5]. Suboptimal stent expansion increases the risk of stent thrombosis and restenosis, ischemic target-lesion revascularization (TLR) and death [5].

Despite their relevance, other calcium-modifying techniques have several limitations [1,4]. Conventional balloons, semi-compliant or NC, are often effective in modifying coronary calcified plaques, and pre-dilating lesions with these balloons frequently prepares the lesion and allows stent implantation with an appropriate minimal stent area [17,18]. However, high-pressure balloon dilatation may expand preferentially to the non-calcified segments of the vessel, delivering insufficient force to induce calcium fracture [1,5]. Moreover, these balloons often lead to dissections between the calcified and the healthy segments [1]. High-pressure dual-layer NC balloons such as the OPN balloon have provided a low-profile device which can exert very high pressure on the lesion with increased uniformity, with an increased risk of coronary dissection or perforation [17,18]. Cutting balloons have microsurgical blades bonded longitudinally along their surface, creating shallow incisions in the calcified atherosclerotic plaque. This exposes the more elastic intimal tissue, allowing for greater vessel compliance and improved stent expansion. These balloons are bulky and present a worse profile to cross the lesion [17,18]. Scoring balloons are semi-compliant balloons with three to four rectangular nitinol-based struts that encircle the balloon in a helical pattern in an attempt to reduce the mechanical trauma on the vessel and present a higher crossing profile [17,18]. Rotational atherectomy uses a diamond-encrusted elliptical burr over a specialized 0.009-inch guidewire and abrades non-elastic, fibro-calcified tissue into small particles while deflecting off softer elastic tissue. A smoother luminal surface is then created, with a luminal diameter increase. Orbital atherectomy, a more recent technique, uses a burr with a diamond-coated crown that ablates the calcified lumen, with a smaller chance of vessel perforation due to the absence of a cutting action. With the use of orbital atherectomy there is continuous blood flow through the artery during the procedure, which potentially reduces the likelihood of slow flow/no reflow [17,18]. Rotational and orbital atherectomy effects are somewhat unpredictable, with little impact on the circumferential calcium [1], and may also produce thermal injury and subsequent platelet activation [5]. Distal calcium embolization with no flow/slow flow, dissection and vessel perforation may also occur with atherectomy techniques [1,4,5]. 

Extracorporeal lithotripsy has been the cornerstone for the treatment of urolithiasis for decades [1,4]. More recently, IVL changed the paradigm of calcified vascular lesions (coronary and peripheral) treatment [1]. The coronary IVL system consists of three components: a generator (that produces an electric impulse), a connector cable and a catheter incorporating the lithotripsy emitters enclosed in a semi-compliant balloon [3]. The catheter, similar to an over-the-wire angioplasty balloon, is available in 2.5–4.0 mm diameter, with a set length of 12 mm [1]. The IVL catheter should be selected at a 1:1 ratio relative to the target-vessel diameter and after a sub-nominal pressure inflation, the emitters produce electric sparks that create vapor bubbles in the surrounding fluid (saline/contrast), that expand and collapse within the balloon, resulting in unfocused acoustic pressure waves that radiate circumferentially and transmurally, with an effective pressure of 50 atmospheres [3,8,19]. This results in multiplane micro/macro fractures in the calcified plaque, that leads to an increase in vascular compliance [1]. OCT analysis has shown a consistent improvement in luminal gain, minimal stent area and stent expansion after IVL [8]. As the IVL emitters generate a circumferential wave, arterial circumferential calcification is probably the most suitable target for this technique [1]. However, there is growing evidence of procedural success in all forms of calcified lesions, including eccentric calcified plaques and calcified nodules [1,8]. The presence of calcified nodules has been reported as a risk factor for poor stent expansion and impaired outcomes, thus requiring adequate and dedicated plaque preparation. However, the optimal strategy remains unclear in this situation [20]. The use of conventional non-compliant balloons could appear as a simple first-line strategy but is frequently inefficient for obtaining a correct pre-stent implantation result [20]. The abrasive tools such as rotational or orbital atherectomy have an uncertain impact on this type of lesion, as they might not correctly prepare the most eccentric portion of the calcified stenosis and could, in addition, damage the healthy portion of the vessel [13,20]. Contrastingly, IVL therapy has been reported to be safe and equally efficient in calcified nodules and non-calcified nodule lesions (according to the post-stenting minimal lumen area measurements) [13,20].

Compared to other calcium-modifying techniques, IVL presents several advantages: the efficiency is powered by acoustic burst and not by high-pressure balloon inflation, which significantly decreases the risk of dissection or more severe complications, namely perforation [1,3,5]. Although both rotational atherectomy (RA) and IVL lead to visible modification of calcified plaques, the mechanisms by which the two techniques modify the calcified plaque are different, and this should be considered when planning the strategy for PCI with severely calcified lesions [21]. RA may be more suitable for lesions with high eccentricity to facilitate subsequent balloon or stent deployment, owing to a greater luminal gain [21]. IVL affects both superficial and deep calcium by inducing more and longer fractures of the calcified plaque, leading to improved vascular compliance [1,3,5,21]. So, IVL may be better suited in lesions with long calcification and calcified plaques with circumferential or nearly circumferential calcification may be better modified by IVL [21]. Moreover, in contrast to atherectomy, IVL decreases the risk of embolization and allows side branch protection with a second guide-wire [1,3,5]. IVL may also be an option to treat acutely underexpanded stents as the sonic waves seem safe in this set and did not significantly affect stent integrity in several in vitro studies [22,23]. There are ongoing head-to-head comparison studies between the use of super high-pressure NC balloons and IVL for the treatment of heavily calcified lesions [24].

Perhaps the most limiting factor of the IVL balloons is their limited crossability profile, far inferior to that of standard balloons. In cases of failure to cross, a hybrid rotational atherectomy approach with subsequent IVL use can lead to both superficial and deep calcium modification, with a successful result [1]. RA allows the treatment of intimal calcium and permits to cross balloons or stents through severe lesions. However, when adequate expansion of the balloons is not achieved after RA, IVL, which is not usually able to cross critical stenosis due to its bulky profile, represents an optimal complementary device [25]. In the present study, 12 patients were submitted to a hybrid approach, mainly in cases of CTO, with a primary effectiveness endpoint of 92%, highlighting the usefulness of this approach.

In addition to its general use in calcified lesions, a specific subset where IVL may be of use is in LM lesions. Given its proximal location, the limited crossability of IVL may be less of an issue. However, the most relevant limitation in this lesion subset pertains to the fact that repeated inflation is necessary for the IVL system, which may not be hemodynamically tolerated by patients. Indeed, the use of IVL in LM lesions can lead to prolonged vessel occlusion to deliver the required energy, leading to severe ischemia [1]. In our subgroup of nine patients submitted to LM PCI with IVL, an immediate success was achieved in all cases, with one in-hospital death due to cardiogenic shock that was already present before PCI. Our results therefore suggest IVL is feasible and useful in LM lesions. Other studies have reported similar results. In a retrospective analysis of 31 patients with obstructive calcified distal LM disease treated with IVL, the target minimal stent area was achieved in 97.3% of stented segments, with no in-hospital MACE [26]. Salazar et al. performed an observation study including consecutive patients with severely calcified LM stenosis, in which the primary endpoint (successful stent delivery and expansion with in-stent residual stenosis < 30%) was achieved in all patients [14]. In this study, a ventricular assistance device was used in four cases [14]. Rola et al. showed the efficacy of IVL in LM PCI in 16 patients, 62.5% of them presenting with an acute coronary syndrome, after the attempt to perform plaque modification with other calcium-modifying techniques [27]. 

Lastly, although there are no robust data to support the off-label indication of IVL to achieve optimal stent expansion after its implantation or in the treatment of stent restenosis, there are several clinical cases in the literature reporting its efficacy [1,28,29,30]. In 6.3% of our cases, IVL was used after stent implantation in order to improve the final angiographic outcome, adding further data to support this indication. 

Acute outcomes after IVL in severely calcified coronary lesions have been very reassuring. Ongoing follow-up will determine whether the favorable short-term outcomes of PCI using IVL in severely calcified lesions will persist in a mid- and long-term basis.

## 5. Limitations

This is a single-center observational study with a limited sample size. Thus, generalization may be limited.

Imaging was used in a minority of patients. This was because of catheter availability constraints during the study period. Thus, no detailed imaging data regarding calcium modification, calcium grading or calcium distribution can be provided.

Furthermore, the primary efficacy endpoint was defined angiographically by visual percentage stenosis estimation according to the first operator, rather than core-lab assessment. Thus, bias in the primary efficacy endpoint assessment cannot be ruled out. 

## 6. Conclusions

This real-world cohort data of a single-center observational study suggests that IVL is an effective and safe technique for the treatment of heavily calcified coronary lesions, including in subgroups with previously limited available data—acute coronary syndromes, left main lesions, hybrid debulking plus lithotripsy approach and in-stent restenosis/post-stent implantation optimization. These findings contribute to the growing body of evidence supporting the use of IVL as an additional valuable and supported technique in the management of challenging calcified coronary lesions.

## Figures and Tables

**Table 1:** Baseline characteristics.

Age, years	72 ± 9
Male	89 (80.2)
Diabetes	62 (55.9)
Systemic arterial hypertension	101 (91)
Dyslipidemia	87 (78.4)
Prior PCI	53 (47.7)
Prior coronary artery bypass grafting	17 (15.3)
Prior stroke	2 (1.8)
Current or former smoker	43 (38.7)
Renal insufficiency (eGFR < 60 mL/min/1.73 m^2^)	45 (40.5)
Renal replacement therapy	8 (7.2)
Clinical presentation	
STEMI	15 (13.5)
NSTEMI	23 (20.7)
UA	10 (9)
CCS	59 (53.2)
VT/VF	1 (0.9)
HF/CS	3 (2.7)

Values are mean ± SD or n (%). CCS: chronic coronary syndrome; eGFR: estimated glomerular filtration rate; HF: heart failure; NSTEMI: non-ST-elevation myocardial infartion; PCI: percutaneous coronary intervention; STEMI: ST-elevation myocardial infartion; UA: unstable angina; VT: ventricular tachycardia; VF: ventricular fibrillation.

**Table 2:** Angiographic characteristics.

Target Vessel	
LM artery	9
LAD	49
Cx	15
RCA	40
Treated vessel	
1	109 (98.2)
2	2 (1.8)
Vessel diameter (mm)	3 ± 0.5
In-stent restenosis	20 (18)
CTO	12 (10.8)
Bifurcation lesion	9 (8.1)
Bifurcation lesion with side branch involvement	5 (4.5)
Syntax score	23 ± 13

Values are mean ± SD or n (%). CTO: chronic total occlusion; Cx: circumflex artery; LM: left main; LAD: left descending artery; RCA: right coronary artery; SD: standard deviation.

**Table 3:** Procedural details.

Total procedure time, min	99.5 (69)
Contrast volume, mL	237.5 (118)
Access	
Radial	70 (63.1)
Femoral	41 (36.9)
Pre-dilation	106 (95.5)
Number of lithotripsy catheters	1.06 ± 0.3
RVD/IVL balloon	1 [0.17]
Number of pulses	80 ± 25
Balloon dilation after IVL use	43 (38.7)
Stent delivery	104 (93.7)
DEB use	7 (6.3)
Number of stents implanted	1.45 ± 0.8
Total stent length, mm	35 (36)
Post-stent dilation with NC balloon	64 (57.7)
IVL after stent implantation	7 (6.3)
IVUS	21 (18.9)
OCT	3 (2.7)

Values are mean ± SD, median [IQR] or n (%). DEB: drug-eluting balloon; IQR: interquartile range; IVL: intravascular lithotripsy; NC: non-compliant; RVD: reference vessel diameter; SD: standard deviation.

**Table 4:** Primary and secondary endpoints.

**Primary endpoints**	
Primary safety endpoint	4 (3.6)
Primary effectiveness endpoint	111 (100)
**Secondary endpoints**	
Procedural success	111 (100)
Peri-procedural complication	4 (3.6)
Minor dissection	4 (3.6)
Major dissection	0
Perfuration	0
Abrupt closure	0
Slow flow/no reflow	0
30-day cardiac death	3 (2.7)
30-day non-cardiac death	0 (0)
30-day MI	1 (0.9)
30-day TVR	1 (0.9)

Values are n (%). MI: myocardial infarction; TVR: target vessel revascularization.

## Data Availability

All data are available upon requirement.

## Abbreviations

CAD	coronary artery disease
CCS	chronic coronary syndrome
CTO	chronic total occlusion
Cx	circumflex artery
DEB	drug-eluting balloon
eGFR	estimated glomerular filtration rate
HF	heart failure
IVL	intravascular lithotripsy
IVUS	intravascular ultrasound
IQR	interquartile range
LAD	left anterior descending artery
LM	left main artery
MACE	major cardiovascular events
MI	myocardial infarction
NC balloon	non-compliant balloon
NSTEMI	non-ST-segment elevation myocardial infarction
OCT	optical coherence tomography
PCI	percutaneous coronary intervention
RA	rotational atherectomy
RCA	right coronary artery
RVD	reference vessel diameter
SC balloon	semi-compliant balloon
SD	standard deviation
STEMI	ST-segment elevation myocardial infarction
TIMI	thrombolysis in myocardial infarction
TLF	target lesion failure
TLR	target lesion revascularization
UA	unstable angina
VF	ventricular fibrillation
VT	ventricular tachycardia
